# An evaluation of computerized adaptive testing for general psychological distress: combining GHQ-12 and Affectometer-2 in an item bank for public mental health research

**DOI:** 10.1186/s12874-016-0158-7

**Published:** 2016-05-20

**Authors:** Jan Stochl, Jan R. Böhnke, Kate E. Pickett, Tim J. Croudace

**Affiliations:** Department of Health Sciences, University of York, York, UK; Department of Psychiatry, University of Cambridge, Cambridge Biomedical Campus, Box 189, Cambridge, CB2 0QQ UK; Department of Kinanthropology, Charles University, Prague, Czech Republic; Hull York Medical School (HYMS), University of York, York, UK; Dundee Centre for Health And Related Research, School of Nursing & Health Sciences, University of Dundee and Academic Health Science Partnership Tayside, Dundee, UK

**Keywords:** Computerized adaptive testing, General Health Questionnaire, Affectometer, Item Response Theory

## Abstract

**Background:**

Recent developments in psychometric modeling and technology allow pooling well-validated items from existing instruments into larger item banks and their deployment through methods of computerized adaptive testing (CAT). Use of item response theory-based bifactor methods and integrative data analysis overcomes barriers in cross-instrument comparison. This paper presents the joint calibration of an item bank for researchers keen to investigate population variations in general psychological distress (GPD).

**Methods:**

Multidimensional item response theory was used on existing health survey data from the Scottish Health Education Population Survey (*n* = 766) to calibrate an item bank consisting of pooled items from the short common mental disorder screen (GHQ-12) and the Affectometer-2 (a measure of “general happiness”). Computer simulation was used to evaluate usefulness and efficacy of its adaptive administration.

**Results:**

A bifactor model capturing variation across a continuum of population distress (while controlling for artefacts due to item wording) was supported. The numbers of items for different required reliabilities in adaptive administration demonstrated promising efficacy of the proposed item bank.

**Conclusions:**

Psychometric modeling of the common dimension captured by more than one instrument offers the potential of adaptive testing for GPD using individually sequenced combinations of existing survey items. The potential for linking other item sets with alternative candidate measures of positive mental health is discussed since an optimal item bank may require even more items than these.

**Electronic supplementary material:**

The online version of this article (doi:10.1186/s12874-016-0158-7) contains supplementary material, which is available to authorized users.

## Background

Assessment of the psychological component of health via rating scales and questionnaires has a long and continuing history. This is exemplified by the work of Goldberg on his General Health Questionnaire (GHQ) item set(s) [1], but also by many others who have worked on questionnaires measuring “general health” [2]. Goldberg’s GHQ instruments are intended to be scored and used as an assessment of risk for common mental disorder(s) and have become established in health care, help seeking and epidemiological studies including national and cross-national surveys. However, there have also been new and influential measures developed for application in this setting, introduced by researchers from the fields of health promotion, positive psychology, and public (mental) health. Consequently, over the past two decades it has become increasingly common for national and international research studies and health surveys to broaden measurement to a wider range of psychological health concepts in populations [3]. This has resulted in multi-faceted definitions and new instrument conventions for fieldwork [4] such that more than one instrument is now likely to be included in health or well-being surveys.

Presently, a number of alternative instruments appear popular. Hence there are choices and opportunities for researchers and survey designers to experiment with different assemblies, subsets and orderings of existing items within and across instruments [5–7]. Our impression is that this has been rare to date and therefore several instruments that may all assess a common construct may exist and have been developed in parallel [8]. If this argument holds, then there may be no need to invent or introduce new items or instruments, as existing item sets might be sufficient or adequate, and already complement each other in this regard. If this is the case, they can be combined in order to achieve accurate and efficient measurement of population level variation in public health research.

We suggest that, over the past decade, too much of the debate about the measurement of well-being has been about specific instruments, i.e. fixed collections of items, not about the items themselves. Instead of looking at whole instruments and correlations between their scores in order to try to gauge their similarity, the use of item response theory (IRT) based models and joint analysis of items (“co-callibration”) [8–10] may be of greater value in advancing understanding and measurement of psychological distress variation (and dimensions). Such activities make it possible to identify useful items, the extent of overlap between instruments and optimal item sets for specific assessment purposes. Even more than that, IRT models can help to support those who might wish to administer assessments in a shorter time, they offer potentially higher face validity for the individual respondents, yet still with a level of precision that is high enough for any given scientific or practical purpose, as befits any particular study or set of surveys. This can be achieved by employing computer-adaptive procedures that do not require researchers to depend on any single specific instrument or measure, but rather to use a broader “pool” of content consisting of a large collection of items calibrated using IRT: a practice that has become known as computerized adaptive testing (CAT) [11]. Since there is potential for most modern surveys to use technologies that allow items to be administered via apps, on mobile devices or through conventional or cloud-based computing platforms, there is no reason why this technology should not be used to its maximum potential, to support adaptive testing ideas in the field of survey research.

In this paper we present such a joint analysis. Our aim is to combine item sets from two instruments (the GHQ-12 and Affectometer-2) and to offer them as an item bank for general psychological distress [12] measurement. The main aim of such an analysis is the quantification of similarities and overlap across all items - as well as their item parameters -  that can be used for further implementation as an “item bank”. Since we will invoke psychometric principles and models that allow for adaptive measurement, we will also emphasize how the measurement error considered under this approach can enhance narratives about lowest permissible measurement precision across individuals.

To this end, we first compared plausible structural models that were derived from the literature for each instrument and then fit an appropriate latent variable model (from the family of IRT models). This approach allowed us to map GHQ-12 and Affectometer-2 items onto a common dimension measured by both instruments. Hence this general psychological distress "factor" (dimension) was defined via bifactor modeling [13]. Based on this model we next assessed inter-item dependencies and the position of the item parameters on the latent continuum to identify which items of the two instruments were possibly exchangeable [14] and would align to one metric.

Building on the previous steps, we then explored the feasibility of administering the joint item-set as a computerized adaptive test drawing on the 52-item bank. In the simulation study we took an additional opportunity to compare different estimation procedures and configurations of the CAT algorithms as well as exploring the number of items that are necessary to reliably assess a general psychological distress factor. In doing so we aimed to meet the measurement and practical needs of public mental health researchers.

## Methods

### Multi-item questionnaires to be jointly calibrated: integrative data analysis approach

Two instruments are introduced as key measures in the dataset chosen for our analysis. We chose instruments for which there is either extensive literature, or interesting items: the former is our justification for using GHQ-12, and the latter for including Affectometer-2.

The 12 - item version of the GHQ is the shortest and probably the most widely used version of the item set originated by Goldberg [15]. GHQ-12 was developed as a brief, paper and pencil assessment of psychological distress, indicative of common mental disorder (CMD). It identifies those exceeding a threshold on the sum score – “screen positives” who are at increased risk of  a current diagnosis of anxiety and/or depression (i.e. CMD). GHQ-12 is best considered as a short form of the GHQ-30, which itself comprises half the items in the original GHQ-60 [15]. The GHQ-30 was intended to be unidimensional and avoided the inclusion of somatic symptoms. Both GHQ-30 and GHQ-12 contain an equal number of positively and negatively phrased items.

The Affectometer-2 is a 40-item scale developed in New Zealand to measure *general happiness* as a sense of well-being based on assessing the balance of positive and negative feelings in recent experience [16]. Its items contain both simple adjectives and phrases. The Affectometer-2 came to the attention of many UK and international audiences, when it was considered as a starting point for the development of a Scottish population well-being indicator. Comparatively little attention had previously been given to the Affectometer-2 within the UK (only one publication by Tennant and co-authors [17]). Part of the motivation for our analysis was to understand its items in the context of the latent continuum of population general psychological distress since they developed historically in different contexts and were aimed at different purposes. Our methods allow novel combinations of items to be scored on a single population construct, a latent factor common to the whole set of items, using the widely exploited modeling approach of bifactor IRT [18–20].

### Response options, response levels, and scoring

In contrast to the GHQ-12, which has four ordinal response levels (for positively worded items: not at all, no more than usual, rather more than usual, much more than usual; for negatively worded items: more than usual, same as usual, less than usual, much less than usual), the Affectometer-2 has five ordinal response levels (not at all, occasionally, some of the time, often, all of the time). Some Affectometer-2 items, as the instrument has a mixture of positive and negative phrasing, needed to be reversed (half of them) to score in the same “morbidity” direction. Negative GHQ-12 items' response levels are already reversed on the paper form and thus their scoring does not need to be reversed. Nonetheless, positive and negative item wording is known to influence responses [13, 21, 22] regardless of reversed scoring of corresponding items. An approach to eliminate this effect is to model its influence as a nuisance (method) factor in factor analysis, for example by using the bifactor model [23] or alternative approaches [24, 25].

### Population samples for empirical item analysis

A dataset of complete GHQ-12 and Affectometer-2 responses was obtained from *n* = 766 individuals who participated in wave 11 (collected in 2006) of the Health Education Population Survey in Scotland (SHEPS) [26]. This figure comprises effectively half of the total SHEPS sample size that year; the other half was administered the Warwick-Edinburgh Mental Well-Being Scale [27]. The long running series of SHEPS in Scotland was started in 1996 and was designed to monitor health-related knowledge, attitudes, behaviors and motivations to change in the adult population in Scotland. The questionnaires are administered using computer assisted personal interviewing (CAPI) in respondents' homes.

### Development of the latent variable measurement model and item calibration

To empirically test the structural integrity of the 52 items in the proposed general psychological distress item bank we used multidimensional IRT modeling with bifactor principles underpinning our analyses. We tested a priori the hypothesis that both GHQ-12 and Affectometer-2 items contribute mainly to the measurement of a single dimension (psychological distress). However, apart from this dominant (general) factor, responses might also be influenced by methodological features such as item wording (as noted earlier half of the items in the GHQ-12 and Affectometer-2 are positively worded and half negatively worded).

**Fig. 1:**
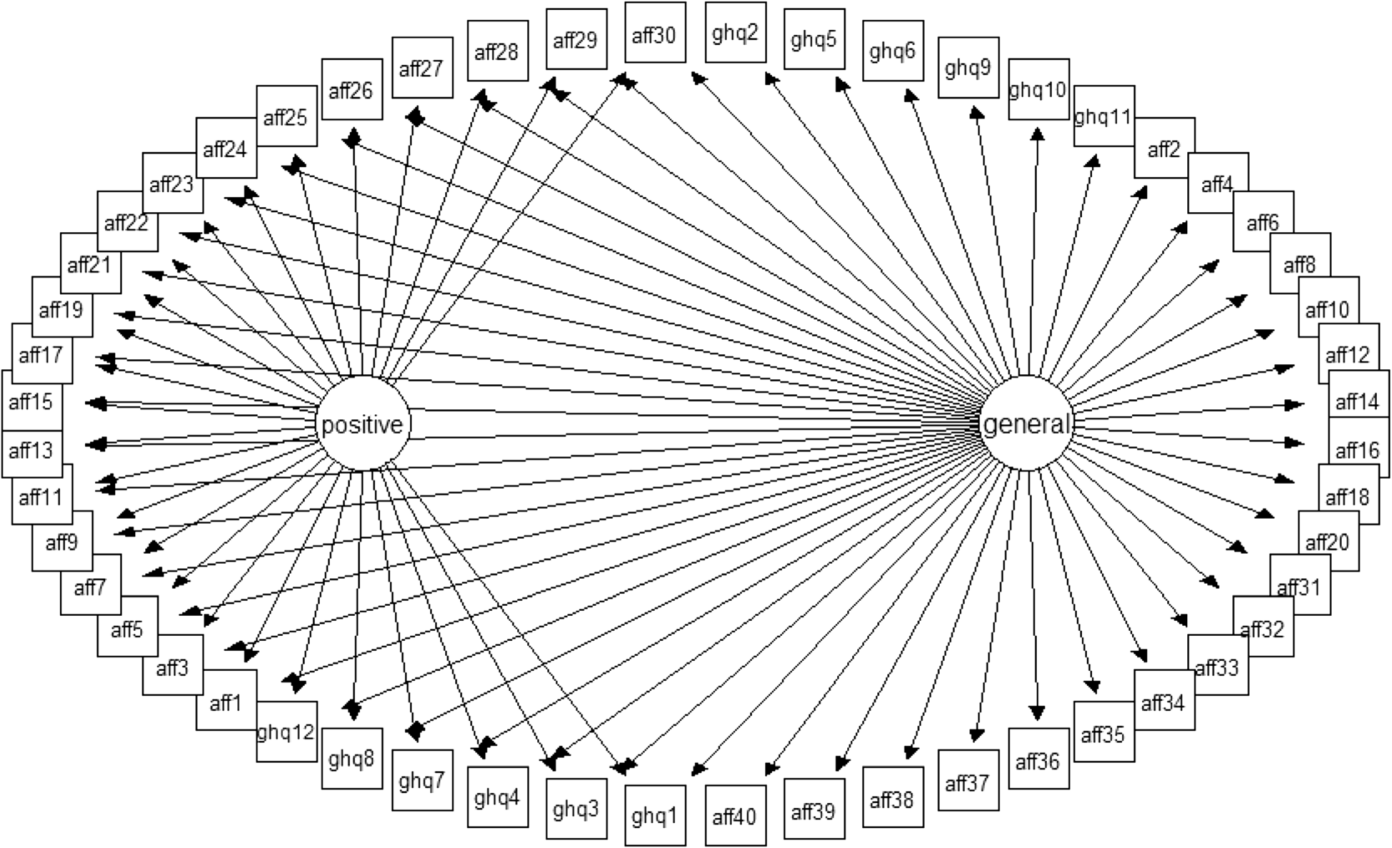
M-1 model of GHQ-12 and Affectometer-2

Several approaches have been suggested to model variance specific to methods factors [24, 25]. To accommodate the possible influences of such item wording effects when seeking the relevant estimates for the main construct of general psychological distress (GPD) we elected to apply a so-called M-1 model [25]. This model assumes the existence of a general factor as well as M-1 method latent variables where M stands for specific (nuisance) factors explaining the common variance of items sharing the same wording. In the framework of our study, the M-1 model translates into the general factor accounting for shared variance (here GPD) across all 52 items in our item bank and one specific factor accounting for positively worded items from both measures^1^. Figure 1 provides a graphical representation of the M-1 model.

To demonstrate the relevance of a bifactor approach for our data, we compare its fit to data with a unidimensional solution, i.e. a solution where all items load on a general factor and no specific factors are included. For evaluation of model fit, traditional fit indices were used, including Satorra-Bentler scaled chi-square [28], comparative fit index (CFI) [29], Tucker-Lewis fit index (TLI) [30] and root mean square error of approximation (RMSEA) [31]. Corrected *χ*^2^ difference test was used for the comparison [32]. All models were estimated with MPlus [33] using mean and variance adjusted Weighted Least Squares (WLSMV) estimation. Therefore the resulting model can be referred as the normal ogive Graded Response Model (GRM) [34, 35].

### CAT simulation

Before the simulation of the adaptive administration of this item bank could be carried out, the factor analytic estimates needed to be converted to IRT parameters by using the following formulas [18, 36]; for each item *i* = 1, … *P* influenced by *m* = 1,…,*M* factors, the discrimination (*α*_*im*_) and *k* IRT thresholds (*t*_*ik*_) on item *i* are

$$ {\alpha}_{im}=\frac{1.7\times {\lambda}_{im}}{\sqrt{1-{\displaystyle \sum_{m=1}^M{\lambda}_{im}^2}}} $$ and $$ {t}_{ik}=\frac{1.7\times {\tau}_{ik}}{\sqrt{1-{\displaystyle \sum_{m=1}^M{\lambda}_{im}^2}}} $$,

where *λ*_*im*_ is factor loading of the item on factor *m*, *τ*_*ik*_ are the corresponding item thresholds and the scaling constant 1.7 converts estimates from the normal ogive metric of the factor model into logistic IRT metric needed for the CAT application.

To evaluate the performance of the proposed item bank we set up a Monte Carlo simulation. The simulation can be used to evaluate the efficacy of CAT administration and also the proximity of the latent factor values from the CAT administration (*θ*_*est*_) to the true latent factor values (*θ*_*true*_). In such a setting, a matrix of item parameter estimates from a calibration study and a vector of values of *θ*_*true*_ need to be provided. Also, the IRT model has to be specified. The process can be outlined as follows:

1. Simulate latent factor values from the desired distribution (*θ*_*true*_) which serve as “true” latent distress values of the simulated respondents.

For the purposes of our simulation we first simulated 10,000 *θ*_*true*_ values from standard normal distribution N(0,1) which is the presumed empirical distribution of distress in the general population. These values are therefore used to investigate the functioning of the item bank in its epidemiological context. We also ran a second simulation based on 10,000 *θ*_*true*_ values drawn from uniform distribution U(-3,3). Although such a distribution of distress is unlikely in the general population, the rationale is to eliminate the influence of the empirical distribution of the latent factor on CAT performance.

2. Supply item parameter estimates and choose the corresponding IRT model.

In the context of our study, this step means to supply IRT parameters (discriminations and item thresholds) from item calibration and define which model was used for the calibration (normal ogive GRM in our case). Together with the *θ*_*true*_ values simulated from the previous step, this provides the information needed for a simulated CAT administration, because stochastic responses to the items can be generated (see step 4).

3. Set CAT administration options

This step involves the selection of a latent factor estimation method, item selection method, termination criteria and other CAT specific settings. It requires careful selection of manipulated options since otherwise the number of cells in the simulation design increases rapidly. In our simulation, we aimed to evaluate the performance of the item bank in combination with the following:

- Latent factor (*θ*) estimators [37]:
  - Maximum likelihood estimation (MLE)
  - Bayesian modal estimation (BME)
  - Expected A Priori estimation (EAP).
- Item selection methods:
  - unweighted Fisher information (UW-FI) [38, 39]
  - pointwise Kullback-Leibler divergence (FP-KL) [40]:

For more details about implementation of these algorithms please see [41]

- Priors for the distribution of *θ* in the population (only for BME and EAP):
  - (standard) normal
  - uniform.
- Termination criteria (whichever comes first): a) standard error of measurement thresholds: 0.25; 0.32; 0.40, 0.45, 0.50 or b) all items are administered.

This resulted in the 50 cells in the simulation design matrix. The following settings were kept constant across all cells:

- Initial *θ* starting values: random draws from U(-1,1)
- Number of items selected for starting portion of CAT: 3
- Number of the most informative items from which the function randomly selects the next item of CAT: 1 (i.e. the most informative item is always selected).

Additional parameters can be added to control the frequency of item selection (indeed most informative items tend to be selected too often and the least informative are selected rarely – this issue is known as item exposure). We do not control for item exposure in our study as it is not considered (yet) to be of great concern in mental health assessment applications, but the simulation study also allowed us to explore the relevance of this aspect for this item bank.

4. Simulate CAT administration

Within each of the cells of the simulation design, an administration of the item bank is simulated for each randomly generated *θ*_*true*_ value (from step 1). Based on an initial starting *θ* value, three items are chosen from the item bank (see step 3, initial *θ* starting values) and stochastic responses are calculated for the respective *θ*_*true*_ values. Based on these responses, an initial estimate of the latent factor value is calculated (see step 3, *θ* estimators); for which a new item to present is selected from the item bank (see step 3, item selection methods). This process is repeated until a pre-set termination criterion is reached (see step 3, termination criteria). This process mimics standard CAT applications [11] and results in estimates (*θ*_*est*_) for each of the simulated *θ*_*true*_.

The CAT simulation analysis was performed in the R package *catIrt* [41]. Please consult its reference manual [41] for a full description of available simulation options. Key information was stored for each simulated CAT administration: which items were administered and their order, estimated *θ*_est_ and its standard error after item administration. Computer code is provided in an Additional file 1.

CAT performance was assessed by means of the number of administered items, mixing of items from GHQ-12 and Affectometer-2 during CAT administration, and by the proximity of *θ*_*est*_ from CAT administration to the simulated *θ*_*true*_. Such proximity can be evaluated based on the root mean squared error, computed as

$$ RMSE=\sqrt{\frac{1}{n}{\displaystyle \sum {\left({\theta}_{est}-{\theta}_{true}\right)}^2}} $$.

Thus, values can be interpreted as the standard deviation of the differences (on the logit scale) between the CAT estimated and the true *θs*. We also present correlations between these two quantities. Lower values of RMSE and correlations closer to unity indicate better performance.

## Results

The left half of Table 1 presents factor loadings and thresholds of the M-1 model. Although *χ*^2^ indicates significant misfit (*χ*^2^ = 4653, df = 1248, *p* < 0.001), other fit indices indicate marginal fit (CFI = 0.922; TLI = 0.917, RMSEA = 0.063). This model showed significant improvement in model fit when compared to the unidimensional solution (*χ*^2^ difference = 948, df = 26, *p* < 0.001).

**Table 1:** Factor loadings (λ) and thresholds (τ) of GHQ-12 and Affectometer-2 items

Item	Abbreviated item wording	M-1 model						Updated model						
		λ gen	λ pos	τ1	τ 2	τ 3	τ 4	λ gen	λ posAff	λ posGHQ	τ1	τ 2	τ 3	τ 4
GHQ 1	Able to concentrate	0.68	−0.12	−1.30	1.01	1.89	-	0.58	-	0.60	−1.30	1.01	1.89	-
GHQ 2	Lost sleep	0.66	-	−0.14	0.87	1.59	-	0.67	-	-	−0.14	0.87	1.59	-
GHQ 3	Play useful part	0.60	−0.14	−0.99	1.24	1.91	-	0.50	-	0.54	−0.99	1.24	1.91	-
GHQ 4	Making decisions	0.65	−0.24	−1.08	1.40	2.14	-	0.52	-	0.61	−1.08	1.40	2.14	-
GHQ 5	Under strain	0.73	-	−0.45	0.76	1.63	-	0.74	-	-	−0.45	0.76	1.63	-
GHQ 6	Overcome difficulties	0.76	-	0.01	1.15	1.75	-	0.77	-	-	0.01	1.15	1.75	-
GHQ 7	Enjoy day-to-day activities	0.65	−0.15	−1.24	0.97	1.75	-	0.56	-	0.49	−1.24	0.97	1.75	-
GHQ 8	Able to face problems	0.64	−0.22	−1.06	1.30	2.04	-	0.50	-	0.68	−1.06	1.30	2.04	-
GHQ 9	Unhappy	0.86	-	0.00	0.93	1.62	-	0.87	-	-	0.00	0.93	1.62	-
GHQ 10	Lose confidence	0.79	-	0.14	1.02	1.77	-	0.81	-	-	0.14	1.02	1.77	-
GHQ 11	Worthless person	0.86	-	0.58	1.38	1.98	-	0.88	-	-	0.58	1.38	1.98	-
GHQ 12	Reasonably happy	0.63	−0.14	−1.01	1.10	1.89	-	0.54	-	0.53	−1.01	1.10	1.89	-
Aff 1	Life on the right track	0.70	0.43	−1.08	0.00	0.53	1.29	0.68	0.46	-	−1.08	0.00	0.53	1.29
Aff 2	Change life	0.67	-	−0.83	0.14	0.74	1.64	0.68	-	-	−0.83	0.14	0.74	1.64
Aff 3	Future looks good	0.62	0.44	−1.27	−0.11	0.48	1.32	0.60	0.47	-	−1.27	−0.11	0.48	1.32
Aff 4	Best years are over	0.63	-	−0.01	0.66	1.16	1.67	0.64	-	-	−0.01	0.66	1.16	1.67
Aff 5	Like yourself	0.48	0.49	−1.01	−0.06	0.57	1.36	0.46	0.50	-	−1.01	−0.06	0.57	1.36
Aff 6	Something wrong	0.77	-	0.27	0.91	1.41	2.10	0.78	-	-	0.27	0.91	1.41	2.10
Aff 7	Handle problems	0.48	0.36	−0.85	0.18	0.75	1.53	0.45	0.40	-	−0.85	0.18	0.75	1.53
Aff 8	Failure	0.88	-	0.39	1.07	1.46	2.22	0.89	-	-	0.39	1.07	1.46	2.22
Aff 9	Loved and trusted	0.53	0.51	−0.45	0.54	1.02	1.64	0.51	0.53	-	−0.45	0.54	1.02	1.64
Aff 10	Left alone	0.61	-	0.28	0.95	1.47	2.22	0.62	-	-	0.28	0.95	1.47	2.22
Aff 11	Close to people	0.52	0.54	−0.48	0.56	0.99	1.81	0.50	0.57	-	−0.48	0.56	0.99	1.81
Aff 12	Lost interest	0.72	-	0.56	1.12	1.77	2.62	0.73	-	-	0.56	1.12	1.77	2.62
Aff 13	Do whatever want	0.49	0.33	−1.26	−0.39	0.28	0.97	0.46	0.37	-	−1.26	−0.39	0.28	0.97
Aff 14	Life stuck	0.81	-	−0.27	0.56	1.04	1.67	0.82	-	-	−0.27	0.56	1.04	1.67
Aff 15	Energy to spare	0.42	0.21	−1.98	−0.92	−0.08	0.83	0.40	0.27	-	−1.98	−0.92	−0.08	0.83
Aff 16	Can’t be bothered	0.66	-	−0.68	0.46	1.08	2.14	0.67	-	-	−0.68	0.46	1.08	2.14
Aff 17	Smiling a lot	0.58	0.30	−1.33	0.04	0.65	1.62	0.56	0.35	-	−1.33	0.04	0.65	1.62
Aff 18	Nothing fun	0.69	-	−0.11	0.80	1.33	2.01	0.70	-	-	−0.11	0.80	1.33	2.01
Aff 19	Thinking creatively	0.53	0.48	−1.19	0.02	0.66	1.58	0.51	0.50	-	−1.19	0.02	0.66	1.58
Aff 20	Thoughts useless	0.76	-	−0.03	0.65	1.27	2.10	0.77	-	-	−0.03	0.65	1.27	2.10
Aff 21	Satisfied	0.66	0.47	−1.37	−0.02	0.60	1.63	0.64	0.50	-	−1.37	−0.02	0.60	1.63
Aff 22	Optimistic	0.44	0.44	−1.44	−0.16	0.43	1.36	0.43	0.43	-	−1.44	−0.16	0.43	1.36
Aff 23	Useful	0.51	0.41	−1.13	0.18	0.82	1.70	0.49	0.45	-	−1.13	0.18	0.82	1.70
Aff 24	Confident	0.62	0.45	−1.17	0.05	0.71	1.59	0.61	0.47	-	−1.17	0.05	0.71	1.59
Aff 25	Understood	0.41	0.41	−1.28	0.01	0.79	1.58	0.40	0.41	-	−1.28	0.01	0.79	1.58
Aff 26	Interested in others	0.40	0.46	−0.77	0.40	0.92	1.74	0.37	0.50	-	−0.77	0.40	0.92	1.74
Aff 27	Relaxed	0.67	0.29	−1.37	−0.10	0.56	1.48	0.66	0.31	-	−1.37	−0.10	0.56	1.48
Aff 28	Enthusiastic	0.55	0.46	−1.51	−0.18	0.50	1.50	0.53	0.50	-	−1.51	−0.18	0.50	1.50
Aff 29	Good natured	0.46	0.45	−0.85	0.47	1.09	2.18	0.43	0.49	-	−0.85	0.47	1.09	2.18
Aff 30	Clear headed	0.53	0.48	−0.86	0.29	0.82	1.67	0.51	0.49	-	−0.86	0.29	0.82	1.67
Aff 31	Discontented	0.73	-	−0.29	0.67	1.27	2.04	0.74	-	-	−0.29	0.67	1.27	2.04
Aff 32	Hopeless	0.86	-	0.47	1.06	1.60	2.26	0.87	-	-	0.47	1.06	1.60	2.26
Aff 33	Insignificant	0.80	-	0.32	1.05	1.59	2.22	0.81	-	-	0.32	1.05	1.59	2.22
Aff 34	Helpless	0.78	-	0.44	1.07	1.55	2.18	0.79	-	-	0.44	1.07	1.55	2.18
Aff 35	Lonely	0.68	-	0.13	0.87	1.26	2.07	0.69	-	-	0.13	0.87	1.26	2.07
Aff 36	Withdrawn	0.83	-	0.23	0.91	1.49	2.26	0.84	-	-	0.23	0.91	1.49	2.26
Aff 37	Tense	0.67	-	−0.72	0.32	0.94	1.83	0.68	-	-	−0.72	0.32	0.94	1.83
Aff 38	Depressed	0.86	-	0.10	0.79	1.25	1.83	0.87	-	-	0.10	0.79	1.25	1.83
Aff 39	Impatient	0.41	-	−1.09	0.16	0.87	2.18	0.41	-	-	−1.09	0.16	0.87	2.18
Aff 40	Confused	0.65	-	0.14	1.00	1.58	2.31	0.66	-	-	0.14	1.00	1.58	2.31

Contrary to what we expected based on the literature, the GHQ-12 positive items did not load on the positive factor (all items show low negative loadings) suggesting that positive items from both instruments do not have much shared variance after accounting for the general factor. Therefore, the updated model considered positively worded items from GHQ-12 and Affectometer-2 (posGHQ and posAff factors respectively) to be separate but correlated factors. The fit to data of this updated model was better compared to the M-1 model (*χ*^2^ = 3135, df = 1247, p < 0.001; CFI = 0.957; TLI = 0.954, RMSEA = 0.047), and direct comparison of both models revealed significant improvement over the M-1 model (*χ*^2^ difference = 321, df = 1, *p* < 0.001). This model was statistically better motivated given the high loadings for the positively worded GHQ-12 items (on the corresponding specific factor). Finally, this model showed better fit in comparison to the unidimensional model (*χ*^2^ difference = 1320, df = 27, *p* < 0.001). Factor loadings and thresholds are presented in the right half of Table 1.

The correlation between the two factors accounting for positively worded items was statistically significant (*p* = 0.003) though small (0.143) suggesting relative independence of the positive wording method factors in GHQ-12 and Affectometer-2. Item loadings for both measures on the general factor were, with the exception of Affectometer-2 item “Interested in others” (Aff 26), all larger than 0.4 which has been suggested as a reasonable cutoff value [42]. This suggests that all covariances of items in our item bank could be explained to a reasonable extent by the single latent factor hypothesized as a population continuum of “general psychological distress”. This interpretation is supported by an *ω*_H_ = .90, which indicates that responses are dominated by this single general factor [18, 36, 43].

**Fig. 2:**
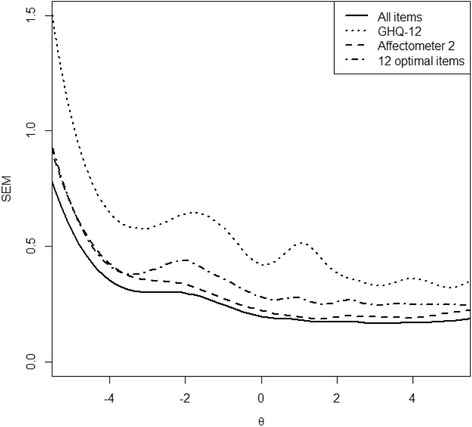
Conditional measurement error for all items, GHQ-12, Affectometer-2, and 12 optimal items from item bank

After the joint calibration on the general factor, it is possible to compare the conditional standard error of measurement (SEM) for the general factor when using either all items or specific subsets of items from the item bank. The comparison of measurement errors of individual instruments revealed that both the GHQ-12 and the Affectometer-2 were best suited to assess more distressed states: Factor estimates above the population mean (“0” in Fig. 2, i.e. more distressed individuals), were associated with a lower standard error of measurement and thus more precisely assessed. The difference between these two item sets was mainly due to their differences in test length as well as the number of response categories (both favour the Affectometer-2). Figure 2 also shows the conditional measurement error for those 12 items from the 52-item bank that are optimally targeted at each distress level to explore whether the item bank improves upon the GHQ-12. In steps of 0.15 along the GPD continuum (x-axis) those 12 items with the highest information function for each specific distress level were selected and their joint information *I*(*θ*) was converted into the conditional measurement error ($1/\sqrt{I\left(\theta \right)}$). The resulting conditional standard error is presented as the dash-dotted line and it illustrates the gain in measurement precision by using items from more than one instrument: in the slightly artificial case of having to choose an optimal 12 item version it is neither the widely relied-upon item set of the GHQ-12 that is chosen, nor is it only Affectometer-2 items with more response categories. Instead, this scenario already illustrates that different items can be of different value for specific assessment purposes and levels of distress. In the following simulation study we assessed this question more generally as well as methodological questions comparing different selection and estimation algorithms for adaptive situations.

The solid line in Fig. 2 shows measurement error along distress levels of the combined instruments. It can also be viewed as a justification for our most stringent termination criteria with respect to SEM in our simulation (see Methods section): SEM values below 0.25 cannot be achieved with this item bank and therefore it makes little sense to include them in the simulation.

### Transformation of factor analytic estimates into relevant IRT parameters

For the final model considered in our item bank, negative items load on the general factor (distress) only but positive items load on both the general as well as one of the method factor (posGHQ and posAff respectively). Therefore, the number of dimensions for negative items is *M* = 1 but for positive items *M* = 2. As noted previously, to eliminate the influence of item wording, we considered and converted IRT estimates only for the general factor in this model (CAT algorithms for item banks where specific factors are deemed to add further substantive information appear elsewhere [44]). Converted IRT estimates of the items included in our bank are presented in Table 2.

**Table 2:** IRT parameter estimates (in logistic metric) of GHQ-12 and Affectometer-2 items

Item	Abbreviated item wording	Nr. of times Administered^a^		α	t_1_	t_2_	t_3_	t_4_
		*θ*_*true*_ ~ N(0,1)	*θ*_*true*_ ~ U(-3,3)					
GHQ 1	Able to concentrate	140	1105	1.75	−3.95	3.08	5.75	-
GHQ 2	Lost sleep	2295	2010	1.53	−0.32	1.99	3.63	-
GHQ 3	Play useful part	865	2627	1.27	−2.50	3.12	4.81	-
GHQ 4	Making decisions	560	2286	1.46	−3.07	3.97	6.08	-
GHQ 5	Under strain	4071	4265	1.87	−1.13	1.93	4.11	-
GHQ 6	Overcome difficulties	4618	2279	2.07	0.04	3.08	4.69	-
GHQ 7	Enjoy day-to-day activities	475	2152	1.42	−3.15	2.47	4.46	-
GHQ 8	Able to face problems	374	1947	1.59	−3.36	4.11	6.46	-
GHQ 9	Unhappy	7578	6138	2.94	0.01	3.17	5.48	-
GHQ 10	Lose confidence	4922	3077	2.31	0.39	2.93	5.07	-
GHQ 11	Worthless person	1055	2449	3.07	2.03	4.84	6.97	-
GHQ 12	Reasonably happy	1007	2757	1.39	−2.60	2.84	4.87	-
Aff 1	Life on the right track	8420	6903	2.01	−3.19	−0.01	1.58	3.80
Aff 2	Change life	3686	4405	1.58	−1.94	0.32	1.71	3.81
Aff 3	Future looks good	3168	4175	1.57	−3.35	−0.29	1.27	3.48
Aff 4	Best years are over	0	19	1.41	−0.02	1.47	2.56	3.70
Aff 5	Like yourself	1287	3031	1.08	−2.35	−0.14	1.33	3.16
Aff 6	Something wrong	3257	3066	2.15	0.74	2.49	3.86	5.77
Aff 7	Handle problems	751	2340	0.96	−1.81	0.38	1.60	3.25
Aff 8	Failure	4134	4193	3.35	1.48	4.03	5.48	8.33
Aff 9	Loved and trusted	1934	2901	1.29	−1.14	1.37	2.57	4.14
Aff 10	Left alone	0	9	1.34	0.60	2.06	3.19	4.81
Aff 11	Close to people	1893	3101	1.29	−1.25	1.47	2.58	4.70
Aff 12	Lost interest	0	8	1.82	1.40	2.79	4.41	6.52
Aff 13	Do whatever want	1016	2794	0.98	−2.65	−0.83	0.59	2.04
Aff 14	Life stuck	7780	8080	2.46	−0.80	1.69	3.12	5.01
Aff 15	Energy to spare	195	952	0.77	−3.84	−1.78	−0.16	1.61
Aff 16	Can’t be bothered	2493	3771	1.54	−1.56	1.04	2.48	4.91
Aff 17	Smiling a lot	1130	2881	1.28	−3.02	0.08	1.47	3.67
Aff 18	Nothing fun	3141	2230	1.67	−0.26	1.90	3.18	4.80
Aff 19	Thinking creatively	1299	3032	1.23	−2.88	0.04	1.60	3.81
Aff 20	Thoughts useless	6031	4153	2.06	−0.09	1.75	3.39	5.62
Aff 21	Satisfied	4655	4218	1.85	−3.98	−0.06	1.73	4.74
Aff 22	Optimistic	587	2307	0.92	−3.08	−0.34	0.93	2.91
Aff 23	Useful	954	2727	1.11	−2.58	0.41	1.86	3.87
Aff 24	Confident	1579	3226	1.61	−3.10	0.13	1.88	4.21
Aff 25	Understood	303	1370	0.83	−2.65	0.03	1.63	3.27
Aff 26	Interested in others	24	132	0.80	−1.66	0.86	1.99	3.75
Aff 27	Relaxed	4299	4703	1.66	−3.42	−0.26	1.41	3.70
Aff 28	Enthusiastic	1719	3348	1.31	−3.76	−0.44	1.24	3.73
Aff 29	Good natured	670	2379	0.97	−1.91	1.06	2.46	4.90
Aff 30	Clear headed	1568	3238	1.24	−2.08	0.70	1.98	4.04
Aff 31	Discontented	4462	3809	1.87	−0.74	1.69	3.22	5.16
Aff 32	Hopeless	2079	3000	3.02	1.63	3.67	5.55	7.84
Aff 33	Insignificant	2923	2417	2.32	0.93	3.02	4.58	6.39
Aff 34	Helpless	2409	2541	2.22	1.23	2.98	4.33	6.08
Aff 35	Lonely	0	9	1.64	0.31	2.04	2.98	4.89
Aff 36	Withdrawn	5565	5027	2.59	0.71	2.82	4.62	7.02
Aff 37	Tense	2763	3937	1.58	−1.67	0.75	2.19	4.24
Aff 38	Depressed	7865	6862	3.00	0.36	2.72	4.30	6.30
Aff 39	Impatient	40	231	0.77	−2.03	0.29	1.62	4.07
Aff 40	Confused	0	10	1.49	0.32	2.27	3.56	5.23

^a^Number of times the items was administered out of 10,000 simulated CAT administration for SEM = 0.32, MLE and UW-FI item selection algorithm

### CAT simulation

We used IRT parameters from Table 2 and a vector of 10,000 values of *θ*_*true*_ sampled from the standard normal and uniform distributions as an input for our simulation. We then manipulated (1) *θ* estimator, (2) item selection method, (3) termination criteria and (4) prior information on distress distribution in the population (for BME and EAP estimators).

To evaluate the efficacy of CAT administration we present the number of administered items needed to reach >the desired termination criteria in Table 3. The results indicate that, to reach a high measurement precision [45, 46] of the score (i.e. standard error of measurement (SEM) = 0.25), 23–30 items on average need to be administered regardless of *θ* estimator, item selection method, or *θ*_*true*_ distribution. Not surprisingly, the number of items needed decreases dramatically as the desired SEM cutoff increases (and thus measurement precision decreases). For example, when the desired SEM cutoff is 0.32, CAT administration requires on average 10–15 items; and only 4–7 items are required for a SEM cutoff of 0.45. It is not surprising that maximum likelihood-based and Bayesian *θ* estimators with non-informative (uniform) priors are similarly effective since they are formally equivalent. However, the normal prior helps to further decrease the number of administered items, even for uniformly distributed *θ*_*true*_ values. Information-based and Kullback-Leibler item selection algorithms are similarly effective.

**Table 3:** Mean (standard deviation) number of administered items

Theta estimator	Item selection	Prior	SEM threshold *θ*_*true*_ ~ N(0,1)					SEM threshold *θ*_*true*_ ~ U(-3,3)				
			0.25	0.32	0.40	0.45	0.50	0.25	0.32	0.40	0.45	0.50
MLE	UW-FI	-	25 (13)	12 (6)	7 (3)	6 (2)	5 (2)	29 (17)	15 (9)	9 (5)	7 (3)	5 (3)
MLE	FP-KL	-	25 (13)	12 (6)	7 (3)	6 (2)	5 (2)	29 (17)	15 (9)	9 (5)	7 (3)	6 (3)
BME	UW-FI	Normal	23 (12)	10 (5)	5 (2)	4 (2)	3 (1)	28 (17)	13 (7)	7 (4)	5 (3)	4 (2)
BME	UW-FI	Uniform	25 (13)	12 (6)	7 (3)	6 (3)	5 (2)	29 (17)	15 (9)	9 (5)	7 (4)	6 (3)
BME	FP-KL	Normal	23 (12)	10 (5)	5 (2)	4 (2)	3 (1)	28 (17)	13 (7)	7 (4)	5 (3)	4 (2)
BME	FP-KL	Uniform	25 (13)	12 (6)	7 (3)	6 (3)	5 (2)	29 (17)	15 (9)	9 (5)	7 (4)	6 (3)
EAP	UW-FI	Normal	23 (12)	11 (5)	6 (2)	5 (2)	4 (1)	28 (17)	13 (7)	7 (4)	5 (3)	4 (2)
EAP	UW-FI	Uniform	26 (13)	13 (6)	8 (3)	6 (2)	5 (2)	30 (17)	15 (9)	9 (4)	7 (3)	6 (2)
EAP	FP-KL	Normal	23 (12)	11 (5)	6 (2)	5 (2)	4 (1)	28 (17)	13 (7)	7 (4)	5 (3)	4 (2)
EAP	FP-KL	Uniform	26 (13)	13 (6)	8 (3)	6 (2)	5 (2)	30 (17)	15 (8)	9 (4)	7 (3)	6 (2)

Table 4 shows the mixing of items from both GHQ-12 and Affectometer-2 when jointly used for CAT administration. Such mixing is relatively stable across all scenarios for high measurement precisions. The variability across scenarios increases with decreasing demands for measurement precision. Note, that the percentage of GHQ-12 items within the item bank was 23.1 %. We emphasize that neither item exposure control nor content balancing was used in our simulations.

**Table 4:** Mean % of GHQ-12 items in the CAT administered items

Theta estimator	Item selection	Prior	SEM threshold *θ*_*true*_ ~ N(0,1)					SEM threshold *θ*_*true*_ ~ U(-3,3)				
			0.25	0.32	0.40	0.45	0.50	0.25	0.32	0.40	0.45	0.50
MLE	UW-FI	-	19.7	23.1	24.2	24.6	23.9	20.7	21.5	25.0	25.8	24.9
MLE	FP-KL	-	19.5	22.9	24.0	24.1	23.8	20.6	21.2	24.4	24.9	24.4
BME	UW-FI	Normal	20.4	24.8	28.1	28.1	32.7	20.7	22.0	24.5	24.9	29.0
BME	UW-FI	Uniform	19.5	22.3	23.0	22.1	20.8	20.6	20.6	23.5	24.1	22.9
BME	FP-KL	Normal	20.2	25.0	28.3	28.6	32.8	20.7	22.0	24.8	25.9	30.3
BME	FP-KL	Uniform	19.3	22.3	22.8	21.8	20.9	20.3	20.9	23.5	23.3	22.3
EAP	UW-FI	Normal	20.1	23.9	26.6	27.8	28.3	20.5	21.4	23.7	24.3	26.4
EAP	UW-FI	Uniform	19.5	22.5	25.0	24.9	26.0	20.7	21.2	25.4	26.8	25.1
EAP	FP-KL	Normal	19.9	24.0	27.1	28.0	29.4	20.5	21.6	24.2	24.6	27.4
EAP	FP-KL	Uniform	19.7	22.1	25.2	23.3	25.8	20.2	21.3	24.8	25.4	25.3

% of GHQ-12 items in the item bank: (12/52)*100 = 23.1 %

Values of RMSE between final *θ* estimates from CAT administration (*θ*_*est*_) and their corresponding values of *θ*_*true*_ are provided in Table 5.

**Table 5:** Root mean square errors (RMSE) between CAT estimated *θ*s and true *θ*s

Theta estimator	Item selection	Prior	SEM threshold *θ*_*true*_ ~ N(0,1)					SEM threshold *θ*_*true*_ ~ U(-3,3)				
			0.25	0.32	0.40	0.45	0.50	0.25	0.32	0.40	0.45	0.50
MLE	UW-FI	-	0.253	0.318	0.401	0.449	0.489	0.266	0.322	0.402	0.457	0.499
MLE	FP-KL	-	0.253	0.319	0.401	0.448	0.488	0.266	0.324	0.402	0.457	0.497
BME	UW-FI	Normal	0.251	0.322	0.407	0.453	0.476	0.279	0.355	0.48	0.558	0.619
BME	UW-FI	Uniform	0.257	0.318	0.401	0.447	0.491	0.266	0.318	0.401	0.451	0.502
BME	FP-KL	Normal	0.251	0.322	0.406	0.448	0.474	0.279	0.355	0.48	0.555	0.619
BME	FP-KL	Uniform	0.259	0.318	0.395	0.44	0.484	0.263	0.322	0.396	0.452	0.491
EAP	UW-FI	Normal	0.247	0.313	0.383	0.429	0.465	0.276	0.345	0.448	0.516	0.575
EAP	UW-FI	Uniform	0.253	0.315	0.383	0.422	0.462	0.261	0.319	0.39	0.43	0.466
EAP	FP-KL	Normal	0.247	0.313	0.383	0.427	0.468	0.276	0.346	0.447	0.512	0.585
EAP	FP-KL	Uniform	0.253	0.315	0.377	0.422	0.463	0.263	0.319	0.385	0.43	0.465

Results show that the square root of mean square deviations between the true and estimated *θ* values lies between 0.247 and 0.619 logit (i.e. between 0.15 and 0.36 standard deviation).

**Fig. 3:**
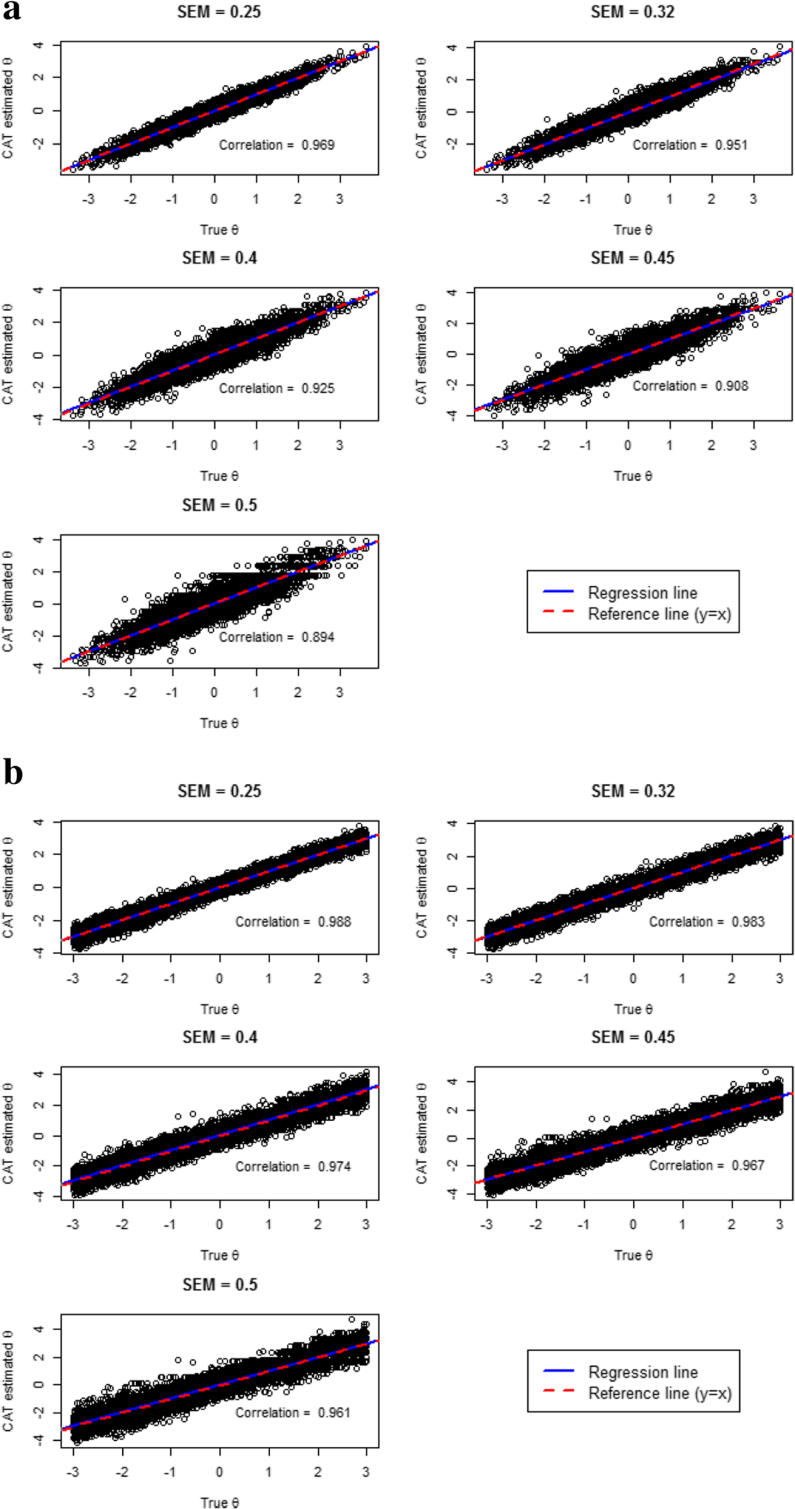
Scatterplots and correlations between CAT estimated *θ*s and true *θ*s for **a**) * θ*_*true*_ ~ N(0,1) and **b**) * θ*_*true*_ ~ U(-3,3)

Another traditional approach for evaluating the proximity of the estimated and true *θs* is the correlation coefficient. Figure 3 therefore provides scatterplots of *θ*_*true*_ on the x-axis and the final estimates *θ*_*est*_ from the CAT administration on the y-axis (for the UW-FI method of item selection).

The red line represents perfect correlation between *θ*_*true*_ and *θ*_*est*_, the blue one shows the fitted regression line. Figure 3 also shows no systematic bias of CAT estimated *θs* for all SEM cutoffs (dots are distributed symmetrically along the red line). As expected, correlation is lower as the measurement precision decreases, though it is still around 0.9 even for a SEM cutoff of 0.50.

## Discussion

The development of an item bank for measurement of psychological distress is a timely challenge amid public mental health debates over measuring happiness /well-being or depression [47–51]. In this paper we have presented, to our knowledge, the first calibration of items to measure GPD “adaptively” focusing on practical issues in the transition from multi-instrument paper and pencil assessments to modern adaptive ones based on item banks created from *existing validated* items. We chose the GHQ-12 and the Affectometer-2 because they are close in terms of content, and target population [16] but were derived differently. We have demonstrated that their items measure a common dimension, which is in keeping with others’ prior notions of general psychological distress. Potentially more instruments targeting the same or similar constructs can be combined to develop large item banks desirable for adaptive testing. Thus, we do not necessarily need to invent new instruments or items - we can instead combine existing and validated ones^2^.

Importantly, the combination of both instruments leads to an item bank which is more efficient than using either instrument on its own. Compared to the GHQ-12, using the same number of items results in a higher measurement precision (dash-dotted  line in Fig. 2) and compared to the Affectometer-2 a smaller number of items will result in sufficient measurement precision for a broad range of distress levels and assessment applications. In addition, although the Affectometer-2 already consists of 40 items, the simulation study (Table 4) shows that the GHQ-12 complements its coverage of the latent construct. These can be seen as considerable advantages over the traditional use of single instruments.

Pooling and calibration of this relatively small set of items required subtle analytic considerations regarding positive wording of items present in both GHQ-12 and Affectometer-2. To eliminate the influence of wording effects on our general factor we used the M-1 modelling approach [25]. A model with a single method factor accounting for the positive wording used by items in both measures was compared to an alternative model with separate method factors for positively worded items in the GHQ-12 and Affectometer-2. Low method factor loadings of GHQ-12 items and only marginal fit of the former model suggest the superiority of the latter model. Interestingly, results show the positive factors from each measure to be relatively independent.

A large literature has considered the potential multidimensionality of the GHQ-12 [52–54]. Usually two correlated factors, one for positive and one for negative items, have been reported. Some authors have interpreted this finding as evidence for the GHQ-12 measuring positive and negative mental health. Others have voiced the concern that the second factor is mostly a methods artifact [55] due to item wording. Our item response theory based factor analysis suggests that it probably is not the former, because if the items of the GHQ-12 and the Affectometer-2 were designed to assess positive mental health with the positively phrased items and mental distress with the negatively phrased ones, then this should be mirrored by a two-factor solution across both instruments. Instead, in our models, GHQ-12 and Affectometer-2 need separate method factors to explain left-over variance in the positively phrased items. This suggests that there is little support for either the same response tendency or the same latent construct underlying the positively worded items across both instruments. This is an important finding, since it indicates first that both instruments, across all their items, assess a single dimension and secondly, that the additional variance in the positively phrased items needs at least two relatively uncorrelated variables as an adequate explanatory model. There is of course interest in exactly what these factors capture, but this is difficult to say without external validation data [8]. It could be, for example, that one of them actually is a pure methods factor, while the other captures a component of positive affect [56, 57]. How relevant this latter question is, remains to be seen, since our results improve further on the current state of this debate: A reliability estimate of *ω*_H_ = .90 for the general psychological distress factor highlights that the systematic variance connected with the positively phrased items of both instruments comprises only a marginal proportion of the total variance in responses.

Most importantly for our purposes here, it is the factor loadings on the general factor from a model with separate method factors for positively worded items that were transformed into IRT parameters to calibrate our general psychological distress (GPD) continuum. These were then used as input for our simulation of the efficacy of CAT administration of this candidate item bank. Depending on the combination of *θ* estimator and item selection method, the average number of items required for CAT administration to reach a SEM cutoff of 0.32 typically required for studies using individual level assessment ranged from 10 to 15. The number of administered items can be further reduced if lower precision is acceptable (see Table 3). These figures show evidence of high efficiency and therefore the usefulness of CAT administration to reduce burden on respondents. However, these results have to be judged within the CAT context and they do not provide information on the number of items needed for a self-report approach to distress assessment with traditional fixed-length questionnaires. The CAT application uses a set of different questions for each respondent optimized for their respective distress levels. Fixed-format questionnaires do not have this flexibility and unless they are targeted at a specific factor level, they probably need to be (much) longer than the results of the CAT simulation indicate [12, 58].

In our simulation we selected frequently used options to show how different combinations of CAT settings may affect the number of administered items. In terms of efficacy, the results suggest rather similar performance of most of them. However, an informative (standard normal) prior helps to further reduce the number of items, especially for lower measurement precisions. Researchers should be cautious when specifying informative priors though, as priors not corresponding with the population distribution may have an adverse effect on the number of administered items [59].

We believe that our argument and technical work are illustrative and compelling as a justification for future fieldwork. However, there are clearly some limitations of our study. It is important to recognize that the simulation may show slightly over-optimistic results in terms of CAT efficiency. This is because the idealized persons’ responses to items during our CAT simulation are based on modelled probabilities and thus follow precisely the item response model used for calibration. Thus the extent of model misfit from the empirical samples is not taken into account by this work. When items are calibrated using a very large sample of respondents, this is not a big issue, but our calibration sample was of only a moderate size and therefore our item bank may need re-calibration in larger empirical datasets. We are not aware of any existing large dataset that allows this, but it could become a priority to explore such a dataset.

An aspect important for future content development is the GPD factor itself. Here, we offer this term over the original terminology (“common mental disorder”) frequently associated with the GHQ because our item bank includes Affectometer-2 items and therefore the measured construct is broader. Looking at the items that have been used in the past, approaches to measure GPD currently range from symptoms of mental disorders, a perspective which overlaps with the GHQ-12 tradition [60–62], to definitions based on the affective evaluation, closer to the underlying rationale of the Affectometer-2 [56, 57]. These, sometimes more deficit oriented perspectives can then be contrasted with similar assessments based on positive psychology or well-being theories [27, 63]. The interrelations of these frameworks are currently under-researched and more integrative research on these is needed [8, 64, 65]. It should be noted that while our analysis presents evidence for overlap between two of these positions, this does not cover all relevant frameworks, nor do we present evidence for predictive or differential validity of the item sets, which would have been beyond the scope of this work.

## Conclusions

The CAT administration of the proposed item bank consisting of GHQ-12 and Affectometer-2 items is more efficient than the use of either measure alone and its use shows a reasonable mixing of items from each of the two measures. The approach outlined in this manuscript combines previous work on data integration and multidimensional IRT, and together with other important and similarly minded developments in the field [66–68] illustrates a possible future of quick and broad assessments in epidemiology and public mental health.

### Ethics approval and consent to participate

Not applicable.

### Consent for publication

Not applicable.

### Availability of data and materials

Data from these secondary data analyses of the SHEPS sample were supplied by the UK Data Archive (study number SN5713) and can be accessed at https://discover.ukdataservice.ac.uk.

## Additional file

Additional file 1: R code for CAT simulation (DOCX 23.7 kb)

## Abbreviations

BME: bayesian modal estimation
CAPI: computer assisted personal interviewing
CAT: computerized adaptive testing
CFI: comparative fit index
CMD: common mental disorder
EAP: Expected A Priori
FP-KL: pointwise Kullback-Leibler divergence
GHQ: General Health Questionnaire
GPD: general psychological distress
GRM: graded response model
IRT: item response theory
MLE: maximum likelihood estimation
RMSE: root mean squared error
RMSEA: root mean square error of approximation
SEM: standard error of measurement
SHEPS: Scottish Health Education Population Survey
TLI: Tucker-Lewis index
UW-FI: unweighted Fisher information
WLSMV: mean and variance adjusted Weighted Least Squares

## Footnotes

1 The selection of modelling a specific factor for negatively or positively worded items is arbitrary and depends on the selection of “reference wording”. We selected the negative wording as our reference type of wording.

2 Subject to considerations regarding copyright permissions.

## References

[1] Goldberg DP, Williams P. A user's guide to the General Health Questionnaire. Windsor UK: NFER-Nelson; 1988.

[2] McDowell I. Measuring health: A guide to rating scales and questionnaires. New York: Oxford University Press; 2006.

[3] Stewart-Brown S, Knifton L, Quinn N (2013). Defining and measuring mental health and wellbeing. Public mental health: global perspectives. edn.

[4] Lindert J, Bain PA, Kubzansky LD, Stein C (2015). Well-being measurement and the WHO health policy Health 2010: systematic review of measurement scales. Eur J Public Health.

[5] Wahl I, Löwe B, Bjorner JB, Fischer F, Langs G, Voderholzer U, Aita SA, Bergemann N, Brähler E, Rose M. Standardization of depression measurement: a common metric was developed for 11 self-report depression measures. J Clin Epidemiol. 2014;67(1):73–86 10.1016/j.jclinepi.2013.04.019 24262771

[6] Weich S, Brugha T, King M, McManus S, Bebbington P, Jenkins R, Cooper C, McBride O, Stewart-Brown S (2011). Mental well-being and mental illness: findings from the Adult Psychiatric Morbidity Survey for England 2007. Br J Psychiatry.

[7] Gibbons RD, Perraillon MC, Kim JB (2014). Item response theory approaches to harmonization and research synthesis. Health Serv Outcomes Res Methodol.

[8] Böhnke JR, Croudace TJ. Calibrating well-being, quality of life and common mental disorder items: psychometric epidemiology in public mental health research. Br J Psychiatry. 2015. doi:10.1192/bjp.bp.115.165530. 10.1192/bjp.bp.115.165530 PMC4967770 26635327

[9] Hussong AM, Curran PJ, Bauer DJ (2013). Integrative data analysis in clinical psychology research. Annu Rev Clin Psychol.

[10] Bauer DJ, Hussong AM (2009). Psychometric approaches for developing commensurate measures across independent studies: traditional and new models. Psychol Methods.

[11] Wainer H, Dorans NJ, Flaugher R, Green BF, Mislevy RJ. Computerized adaptive testing: A primer. Hillsdale, NJ: Lawrence Erlbaum; 2000.

[12] Böhnke JR, Lutz W (2014). Using item and test information to optimize targeted assessments of psychological distress. Assessment.

[13] Hankins M (2008). The factor structure of the twelve item General Health Questionnaire (GHQ-12): The result of negative phrasing?. Clin Pract Epidemiol Ment Health.

[14] Egberink IJL, Meijer RR (2011). An item response theory analysis of Harter’s Self-Perception Profile for children or why strong clinical scales should be distrusted. Assessment.

[15] Goldberg DP (1972). The detection of psychiatric illness by questionnaire.

[16] Kammann R, Flett R (1983). Affectometer 2: A scale to measure current level of general happiness. Aust J Psychol.

[17] Tennant R, Joseph S, Stewart-Brown S (2007). The Affectometer 2: a measure of positive mental health in UK populations. Qual Life Res.

[18] Reise SP (2012). The rediscovery of bifactor measurement models. Multivar Behav Res.

[19] Gibbons RD, Bock RD, Hedeker D, Weiss DJ, Segawa E, Bhaumik DK, Kupfer DJ, Frank E, Grochocinski VJ, Stover A (2007). Full-Information item bifactor analysis of graded response data. Appl Psych Meas.

[20] Gibbons R, Hedeker D (1992). Full-information item bi-factor analysis. Psychometrika.

[21] Romppel M, Braehler E, Roth M, Glaesmer H (2013). What is the General Health Questionnaire-12 assessing?: Dimensionality and psychometric properties of the General Health Questionnaire-12 in a large scale German population sample. Compr Psychiatry.

[22] Ye S (2009). Factor structure of the General Health Questionnaire (GHQ-12): The role of wording effects. Pers Indiv Differ.

[23] Wang W-C, Chen H-F, Jin K-Y. Item response theory models for wording effects in mixed-format scales. Educ Psychol Meas. 2014;75(1):157-78. 10.1177/0013164414528209 PMC5965508 29795817

[24] Pohl S, Steyer R (2010). Modeling common traits and method effects in multitrait-multimethod analysis. Multivar Behav Res.

[25] Geiser C, Lockhart G (2012). A comparison of four approaches to account for method effects in latent state–trait analyses. Psychol Methods.

[26] Scotland NH (2006). Health Education Population Survey.

[27] Tennant R, Hiller L, Fishwick R, Platt S, Joseph S, Weich S, Parkinson J, Secker J, Stewart-Brown S (2007). The Warwick-Edinburgh Mental Well-being Scale (WEMWBS): development and UK validation. Health Qual Life Outcomes.

[28] Satorra A, Bentler PM, von Eye A, Clogg CC (1994). Corrections to test statistics and standard errors in covariance structure analysis. Latent variables analysis: Applications for developmental research. edn.

[29] Bentler PM (1990). Comparative fit indexes in structural models. Psychol Bull.

[30] Tucker LR, Lewis C (1973). A reliability coeffficient for maximum likelihood factor analysis. Psychometrika.

[31] Steiger JH, Lind J. Statistically-based tests for the number of common factors. Paper presented at the annual Spring Meeting of the Psychometric Society in Iowa City. May 30, 1980.

[32] Satorra A, Heijmans RDH, Pollock DSG, Satorra A (2000). Scaled and adjusted restricted tests in multi-sample analysis of moment structures. Innovations in multivariate statistical analysis A Festschrift for Heinz Neudecker. edn.

[33] Muthén L, Muthén B. Mplus: Statistical analysis with latent variables. Version 7.3. Los Angeles, CA: Muthén & Muthén; 1998-2016.

[34] Samejima F (1969). Estimation of latent ability using a response pattern of graded scores.

[35] Takane Y, Leeuw J (1987). On the relationships between item response theory and factor analysis of discretized variables. Psychometrika.

[36] McDonald RP (1999). Test theory: A unified treatment.

[37] Baker FB, Kim SH (2004). Item response theory: Parameter estimation techniques.

[38] Veerkamp WJ, Berger MP (1997). Some new item selection criteria for adaptive testing. J Educ Behav Stat.

[39] van der Linden W (1998). Bayesian item selection criteria for adaptive testing. Psychometrika.

[40] Chang H-H, Ying Z (1996). A global information approach to computerized adaptive testing. Appl Psych Meas.

[41] Nydick SW: catIrt: An R package for simulating IRT-based computerized adaptive tests. R package version 0.4-2. http://CRAN.R-project.org/package=catIrt. In*.*; 2014.

[42] Fliege H, Becker J, Walter OB, Bjorner JB, Klapp BF, Rose M (2005). Development of a computer-adaptive test for depression (D-CAT). Qual Life Res.

[43] Zinbarg R, Revelle W, Yovel I, Li W (2005). Cronbach’s α, Revelle’s β, and Mcdonald’s ωH: their relations with each other and two alternative conceptualizations of reliability. Psychometrika.

[44] Weiss DJ, Gibbons RD (2007). Computerized adaptive testing with the bifactor model. Proceedings of the 2007 GMAC Conference on Computerized Adaptive Testing: 2007.

[45] Dimitrov DM (2003). Marginal true-score measures and reliability for binary items as a function of their IRT parameters. Appl Psych Meas.

[46] Green BF, Bock RD, Humphreys LG, Linn RL, Reckase MD (1984). Technical guidelines for assessing computerized adaptive tests. J Educ Meas.

[47] Seligman ME, Steen TA, Park N, Peterson C (2005). Positive psychology progress: empirical validation of interventions. Am Psychol.

[48] Ryff CD (1989). Happiness is everything, or is it? Explorations on the meaning of psychological well-being. J Pers Soc Psychol.

[49] Wood AM, Taylor PJ, Joseph S (2010). Does the CES-D measure a continuum from depression to happiness? Comparing substantive and artifactual models. Psychiatry Res.

[50] Joseph S, Lewis CA (1998). The Depression–Happiness Scale: Reliability and validity of a bipolar self‐report scale. J Clin Psychol.

[51] Kammann R, Farry M, Herbison P (1984). The analysis and measurement of happiness as a sense of well-being. Soc Indic Res.

[52] Shevlin M, Adamson G (2005). Alternative factor models and factorial invariance of the GHQ-12: a large sample analysis using confirmatory factor analysis. Psychol Assess.

[53] Werneke U, Goldberg DP, Yalcin I, Ustun BT (2000). The stability of the factor structure of the General Health Questionnaire. Psychol Med.

[54] Hu Y, Stewart-Brown S, Twigg L, Weich S (2007). Can the 12-item General Health Questionnaire be used to measure positive mental health?. Psychol Med.

[55] Molina JG, Rodrigo MF, Losilla JM, Vives J (2014). Wording effects and the factor structure of the 12-item General Health Questionnaire (GHQ-12). Psychol Assess.

[56] Crawford JR, Henry JD (2004). The positive and negative affect schedule (PANAS): construct validity, measurement properties and normative data in a large non-clinical sample. Br J Clin Psychol.

[57] Simms LJ, Gros DF, Watson D, O’Hara MW (2008). Parsing the general and specific components of depression and anxiety with bifactor modeling. Depress Anxiety.

[58] Emons WHM, Sijtsma K, Meijer RR (2007). On the consistency of individual classification using short scales. Psychol Methods.

[59] van der Linden WJ, Glas CAW (2010). Elements of adaptive testing.

[60] Urban R, Kun B, Farkas J, Paksi B, Kokonyei G, Unoka Z, Felvinczi K, Olah A, Demetrovics Z (2014). Bifactor structural model of symptom checklists: SCL-90-R and Brief Symptom Inventory (BSI) in a non-clinical community sample. Psychiatry Res.

[61] Glaesmer H, Braehler E, Grande G, Hinz A, Petermann F, Romppel M (2014). The German version of the Hopkins Symptoms Checklist-25 (HSCL-25): Factorial structure, psychometric properties, and population-based norms. Compr Psychiatry.

[62] Stochl J, Khandaker GM, Lewis G, Perez J, Goodyer IM, Zammit S, Sullivan S, Croudace TJ, Jones PB (2015). Mood, anxiety and psychotic phenomena measure a common psychopathological factor. Psychol Med.

[63] Jovanović V (2015). Structural validity of the Mental Health Continuum-Short Form: The bifactor model of emotional, social and psychological well-being. Pers Indiv Differ.

[64] Camfield L, Skevington SM (2008). On subjective well-being and quality of life. J Health Psychol.

[65] Wood AM, Tarrier N (2010). Positive Clinical Psychology: a new vision and strategy for integrated research and practice. Clin Psychol Rev.

[66] Gibbons RD, Weiss DJ, Pilkonis PA, Frank E, Moore T, Kim JB, Kupfer DJ (2012). Development of a computerized adaptive test for depression. Arch Gen Psychiatry.

[67] Gibbons RD, Weiss DJ, Kupfer DJ, Frank E, Fagiolini A, Grochocinski VJ, Bhaumik DK, Stover A, Bock RD, Immekus JC (2008). Using computerized adaptive testing to reduce the burden of mental health assessment. Psych Serv.

[68] Gibbons RD, Weiss DJ, Pilkonis PA, Frank E, Moore T, Kim JB, Kupfer DJ (2014). Development of the CAT-ANX: a computerized adaptive test for anxiety. Am J Psychiatry.

