# Supplementary material for: An evaluation of computerized adaptive testing for general psychological distress: combining GHQ-12 and Affectometer-2 in an item bank for public mental health research
Source: BMC Med Res Methodol. 2016 May 20;16:58. doi: 10.1186/s12874-016-0158-7 (PMC4875708; doi:10.1186/s12874-016-0158-7)
Supplement: Additional file 1: — R code for CAT simulation (DOCX 23.7 kb) [file 12874_2016_158_MOESM1_ESM.docx]

**Appendix 1: R code for CAT simulation**

#### Note that object catirtparamaff refers to table of IRT parameters and object dataghgaff to data matrix #####

#### coinsisting of responses to GHQ and Affectometer 2 items ########

require(catIrt) #package version 0.4.2

simtheta <- runif(10000, min = -3, max = 3) # for uniform distribution of θs or simtheta <- rnorm(n, mean = 0, sd = 1) for normal distribution of θs

catghqsimresp <- array(data = NA, dim=c(10,23,5), dimnames = list(NULL,c("thetaEstimator","ItemSelectionMethod","prior.density","mean.nr.items","SD.nr.items","min.nr.items","max.nr.items","mean.sem","SD.sem","min.sem","max.sem","mean.theta","SD.theta","min.theta","max.theta","mean.true.theta","SD.true.theta","min.true.theta","max.true.theta","nr.termination.fixed","nr.termination.precision","cor.CATtheta.TRUEtheta","MSE"),NULL))

thetaEstimator <- c("MLE","BME","EAP")

ItemSelectionMethod <- c("UW-FI","FP-KL")

stopthreshold <- c(0.25,0.32, 0.40,0.45,0.50)

counter <- 0

pb <- winProgressBar("Overall simulation progress", "%", min=0, max=10*5, initial=0) #max is number of all catirt runs (10 rows, 5 reiabilities cutoff)

for (z in length(stopthreshold)) {

rownumber <- 1

for (i in c(1:length(thetaEstimator))) {

for (j in c(1:length(ItemSelectionMethod))) {

counter <- counter+1

catghqsimresp[rownumber,1,z] <- thetaEstimator[i]

catghqsimresp[rownumber,2,z] <- ItemSelectionMethod[j]

set.seed(1234)

if (thetaEstimator[i]=="MLE" | thetaEstimator[i]=="WLE") {

catobject <- catIrt(catirtparam, mod = "grm", resp=NULL, theta = simtheta, catStart = list(init.theta = runif(10000, min=-1, max=1), n.start = 3, select = ItemSelectionMethod[j], delta=0.1, at = "theta", n.select = 1, score=if(thetaEstimator[i]=="MLE") {"step"} else {thetaEstimator[i]}, step.size = 3, leave.after.MLE = TRUE), catMiddle = list(select = ItemSelectionMethod[j], at = "theta", n.select = 1, delta=0.1, score = thetaEstimator[i], range = c(-6, 6), expos = "none"),catTerm = list(term = c("fixed", "precision"), n.min = 3, n.max = ncol(dataghgaff), score = thetaEstimator[i], p.term = list(method = "threshold",crit=stopthreshold[z])),ddist = dnorm, progress=TRUE)

catobject2 <- summary(catobject)

catobjectindiv <- catobject$cat_indiv

itemmatrix <- matrix(data = NA, nrow = 10000, ncol = ncol(dataghgaff), byrow = FALSE, dimnames = list(paste("person", seq(1,10000),sep=""),paste("sel_item_nr", seq(1,ncol(dataghgaff),sep=""))))

for (k in 1:10000) {itemmatrix[k,1:length(catobjectindiv[[k]]$cat_it)] <- catobjectindiv[[k]]$cat_it}

if (z==1 & i==1 & j==1) {catitems <- itemmatrix} else {catitems <- abind(catitems, itemmatrix, along = 3)

dimnames(catitems)[[3]][counter] <- paste(thetaEstimator[i],ItemSelectionMethod[j],stopthreshold[z],sep="")}

if (z==1 & i==1 & j==1) {catthetabaseline <- catobject$cat_theta

catsembaseline <- catobject$cat_sem

truethetabaseline <- catobject$true_theta

truesembaseline <- catobject$true_sem

totthetabaseline <- catobject$tot_theta

totsembaseline <- catobject$tot_sem

catlengthbaseline <- catobject$cat_length

catinfobaseline <- catobject$cat_info

totinfobaseline <- catobject$tot_info} else { catthetabaseline <- cbind(catthetabaseline,assign(paste(thetaEstimator[i],ItemSelectionMethod[j],stopthreshold[z],sep=""), catobject$cat_theta))

catsembaseline <- cbind(catsembaseline,assign(paste(thetaEstimator[i],ItemSelectionMethod[j],stopthreshold[z],sep=""), catobject$cat_sem))

totthetabaseline <- cbind(totthetabaseline,assign(paste(thetaEstimator[i],ItemSelectionMethod[j],stopthreshold[z],sep=""), catobject$tot_theta))

totsembaseline <- cbind(totsembaseline,assign(paste(thetaEstimator[i],ItemSelectionMethod[j],stopthreshold[z],sep=""), catobject$tot_sem))

catlengthbaseline <- cbind(catlengthbaseline,assign(paste(thetaEstimator[i],ItemSelectionMethod[j],stopthreshold[z],sep=""), catobject$cat_length))

catinfobaseline <- cbind(catinfobaseline,assign(paste(thetaEstimator[i],ItemSelectionMethod[j],stopthreshold[z],sep=""), catobject$cat_info))

totinfobaseline <- cbind(totinfobaseline,assign(paste(thetaEstimator[i],ItemSelectionMethod[j],stopthreshold[z],sep=""), catobject$tot_info))}

catghqsimresp[rownumber,4:7,z] <- round(catobject2$items$sum,digits=3)

catghqsimresp[rownumber,8:11,z] <- round(catobject2$sem$sum[which(row.names(catobject2$sem$sum)=="cat"),], digits=3)

catghqsimresp[rownumber,12:15,z] <- round(catobject2$theta$sum[which(row.names(catobject2$theta$sum)=="cat"),], digits=3)

catghqsimresp[rownumber,16:19,z] <- round(catobject2$theta$sum[which(row.names(catobject2$theta$sum)=="tot"),], digits=3)

catghqsimresp[rownumber,20,z] <- sum(catobject$cat_term=="total")

catghqsimresp[rownumber,21,z] <- sum(catobject$cat_term=="se_thresh")

#catghqsimresp[rownumber,22,z] <- sum(catobject$cat_term=="fi_change")

catghqsimresp[rownumber,22,z] <- round(cor(catobject$cat_theta, catobject$true_theta, use = "pairwise.complete.obs", method = "pearson"), digits=3)

catghqsimresp[rownumber,23,z] <- round(mean((catobject$cat_theta-catobject$true_theta)^2, na.rm=FALSE), digits=3)

print(catghqsimresp[,,z])

rownumber <- rownumber+1

info <- sprintf("%d%% done", round((counter/(10*5)*100)))

setWinProgressBar(pb, counter, sprintf("Overall simulation progress"), info)}

if (thetaEstimator[i]=="BME" | thetaEstimator[i]=="EAP") {

catobject <- catIrt(catirtparam, mod = "grm", resp=NULL, theta = simtheta, catStart = list(init.theta = runif(10000, min=-1, max=1), n.start = 3, select = ItemSelectionMethod[j], delta=0.1, at = "theta", n.select = 1, score=thetaEstimator[i], step.size = 3, leave.after.MLE = TRUE), catMiddle = list(select = ItemSelectionMethod[j], at = "theta", n.select = 1, delta=0.1, score = thetaEstimator[i], range = c(-6, 6), expos = "none"),catTerm = list(term = c("fixed", "precision"), n.min = 3, n.max = ncol(dataghgaff), score = thetaEstimator[i], p.term = list(method = "threshold",crit=stopthreshold[z])),ddist = dnorm, progress=TRUE)

catobject2 <- summary(catobject)

catobjectindiv <- catobject$cat_indiv

itemmatrix <- matrix(data = NA, nrow = 10000, ncol = ncol(dataghgaff), byrow = FALSE, dimnames = list(paste("person", seq(1,10000),sep=""),paste("sel_item_nr", seq(1,ncol(dataghgaff),sep=""))))

for (k in 1:10000) {itemmatrix[k,1:length(catobjectindiv[[k]]$cat_it)] <- catobjectindiv[[k]]$cat_it}

catitems <- abind(catitems, itemmatrix, along = 3)

dimnames(catitems)[[3]][counter] <- paste(thetaEstimator[i],ItemSelectionMethod[j],"normal",stopthreshold[z],sep="")

counter <-counter+1

catthetabaseline <- cbind(catthetabaseline,assign(paste(thetaEstimator[i],ItemSelectionMethod[j],stopthreshold[z], "normal",sep=""), catobject$cat_theta))

catsembaseline <- cbind(catsembaseline,assign(paste(thetaEstimator[i],ItemSelectionMethod[j],stopthreshold[z], "normal",sep=""), catobject$cat_sem))

totthetabaseline <- cbind(totthetabaseline,assign(paste(thetaEstimator[i],ItemSelectionMethod[j],stopthreshold[z], "normal",sep=""), catobject$tot_theta))

totsembaseline <- cbind(totsembaseline,assign(paste(thetaEstimator[i],ItemSelectionMethod[j],stopthreshold[z], "normal",sep=""), catobject$tot_sem))

catlengthbaseline <- cbind(catlengthbaseline,assign(paste(thetaEstimator[i],ItemSelectionMethod[j],stopthreshold[z], "normal",sep=""), catobject$cat_length))

catinfobaseline <- cbind(catinfobaseline,assign(paste(thetaEstimator[i],ItemSelectionMethod[j],stopthreshold[z], "normal",sep=""),catobject$cat_info))

totinfobaseline <- cbind(totinfobaseline,assign(paste(thetaEstimator[i],ItemSelectionMethod[j],stopthreshold[z], "normal",sep=""),catobject$tot_info))

catghqsimresp[rownumber,3,z] <- "normal"

catghqsimresp[rownumber,4:7,z] <- round(catobject2$items$sum,digits=3)

catghqsimresp[rownumber,8:11,z] <- round(catobject2$sem$sum[which(row.names(catobject2$sem$sum)=="cat"),], digits=3)

catghqsimresp[rownumber,12:15,z] <- round(catobject2$theta$sum[which(row.names(catobject2$theta$sum)=="cat"),], digits=3)

catghqsimresp[rownumber,16:19,z] <- round(catobject2$theta$sum[which(row.names(catobject2$theta$sum)=="tot"),], digits=3)

catghqsimresp[rownumber,20,z] <- sum(catobject$cat_term=="total")

catghqsimresp[rownumber,21,z] <- sum(catobject$cat_term=="se_thresh")

#catghqsimresp[rownumber,22,z] <- sum(catobject$cat_term=="fi_change")

catghqsimresp[rownumber,22,z] <- round(cor(catobject$cat_theta, catobject$true_theta, use = "pairwise.complete.obs", method = "pearson"), digits=3)

catghqsimresp[rownumber,23,z] <- round(mean((catobject$cat_theta-catobject$true_theta)^2, na.rm=FALSE), digits=3)

print(catghqsimresp[,,z])

rownumber <- rownumber+1

info <- sprintf("%d%% done", round((counter/(10*5)*100)))

setWinProgressBar(pb, counter, sprintf("Overall simulation progress"), info)

catghqsimresp[rownumber,1,z] <- thetaEstimator[i]

catghqsimresp[rownumber,2,z] <- ItemSelectionMethod[j]

catobject <- catIrt(catirtparam, mod = "grm", resp=NULL, theta = simtheta, catStart = list(init.theta = runif(10000, min=-1, max=1), n.start = 3, select = ItemSelectionMethod[j], delta=0.1, at = "theta", n.select = 1, score=thetaEstimator[i], step.size = 3, leave.after.MLE = TRUE), catMiddle = list(select = ItemSelectionMethod[j], at = "theta", n.select = 1, delta=0.1, score = thetaEstimator[i], range = c(-6, 6), expos = "none"),catTerm = list(term = c("fixed", "precision"), n.min = 3, n.max = ncol(dataghgaff), score = thetaEstimator[i], p.term = list(method = "threshold",crit=stopthreshold[z])),ddist = dunif2, progress=TRUE)

catobject2 <- summary(catobject)

catobjectindiv <- catobject$cat_indiv

itemmatrix <- matrix(data = NA, nrow = 10000, ncol = ncol(dataghgaff), byrow = FALSE, dimnames = list(paste("person", seq(1,10000),sep=""),paste("sel_item_nr", seq(1,ncol(dataghgaff),sep=""))))

for (k in 1:10000) {itemmatrix[k,1:length(catobjectindiv[[k]]$cat_it)] <- catobjectindiv[[k]]$cat_it}

catitems <- abind(catitems, itemmatrix, along = 3)

dimnames(catitems)[[3]][counter] <- paste(thetaEstimator[i],ItemSelectionMethod[j],"uniform",stopthreshold[z],sep="")

catthetabaseline <- cbind(catthetabaseline,assign(paste(thetaEstimator[i],ItemSelectionMethod[j],stopthreshold[z], "uniform",sep=""), catobject$cat_theta))

catsembaseline <- cbind(catsembaseline,assign(paste(thetaEstimator[i],ItemSelectionMethod[j],stopthreshold[z], "uniform",sep=""), catobject$cat_sem))

totthetabaseline <- cbind(totthetabaseline,assign(paste(thetaEstimator[i],ItemSelectionMethod[j],stopthreshold[z], "uniform",sep=""), catobject$tot_theta))

totsembaseline <- cbind(totsembaseline,assign(paste(thetaEstimator[i],ItemSelectionMethod[j],stopthreshold[z], "uniform",sep=""), catobject$tot_sem))

catlengthbaseline <- cbind(catlengthbaseline,assign(paste(thetaEstimator[i],ItemSelectionMethod[j],stopthreshold[z], "uniform",sep=""), catobject$cat_length))

catinfobaseline <- cbind(catinfobaseline,assign(paste(thetaEstimator[i],ItemSelectionMethod[j],stopthreshold[z], "uniform",sep=""),catobject$cat_info))

totinfobaseline <- cbind(totinfobaseline,assign(paste(thetaEstimator[i],ItemSelectionMethod[j],stopthreshold[z], "uniform",sep=""),catobject$tot_info))

catghqsimresp[rownumber,3,z] <- "uniform"

catghqsimresp[rownumber,4:7,z] <- round(catobject2$items$sum,digits=3)

catghqsimresp[rownumber,8:11,z] <- round(catobject2$sem$sum[which(row.names(catobject2$sem$sum)=="cat"),], digits=3)

catghqsimresp[rownumber,12:15,z] <- round(catobject2$theta$sum[which(row.names(catobject2$theta$sum)=="cat"),], digits=3)

catghqsimresp[rownumber,16:19,z] <- round(catobject2$theta$sum[which(row.names(catobject2$theta$sum)=="tot"),], digits=3)

catghqsimresp[rownumber,20,z] <- sum(catobject$cat_term=="total")

catghqsimresp[rownumber,21,z] <- sum(catobject$cat_term=="se_thresh")

#catghqsimresp[rownumber,22,z] <- sum(catobject$cat_term=="fi_change")

catghqsimresp[rownumber,22,z] <- round(cor(catobject$cat_theta, catobject$true_theta, use = "pairwise.complete.obs", method = "pearson"), digits=3)

catghqsimresp[rownumber,23,z] <- round(mean((catobject$cat_theta-catobject$true_theta)^2, na.rm=FALSE), digits=3)

print(catghqsimresp[,,z])

rownumber <- rownumber+1

info <- sprintf("%d%% done", round((counter/(10*5)*100)))

setWinProgressBar(pb, counter, sprintf("Overall simulation progress"), info)}}}}

close(pb)
